# Pulmonary artery aneurysm caused by infective endarteritis attributed to patent ductus arteriosus in children: a case report and literature review

**DOI:** 10.3389/fped.2023.1181462

**Published:** 2023-07-17

**Authors:** Pengpeng Wu, Chao Zheng, Feng Zhang, Pingsheng Wang, Haiyong Zhang, Gang Chen

**Affiliations:** ^1^ Department of Pediatric Cardiothoracic Surgery, Anhui Provincial Children’s Hospital, Anhui, China; ^2^ Pediatric Cardiovascular Center, Children’s Hospital, Fudan University, Shanghai, China

**Keywords:** pulmonary artery aneurysm, infective endarteritis, patent ductus arteriosus, surgical repair, children

## Abstract

We report a case of a 10-year-old male patient with pulmonary artery aneurysm (PAA) caused by infective endarteritis of the pulmonary artery attributed to patent ductus arteriosus. He was found to have patent ductus arteriosus at the age of 2, but he was not treated because of the absence of symptoms and normal physical development. He sought medical attention for fever and cough in August 2022. Echocardiography showed pulmonary artery aneurysm, intrapulmonary artery bulge, patent ductus arteriosus, and pericardial effusion. Contrast-enhanced CT showed pulmonary artery aneurysm, patent ductus arteriosus, and a slight compression of the left main bronchus. Surgery was performed to reconstruct the main pulmonary trunk and repair the ductus arteriosus in November 2022. The surgical outcomes were satisfactory.

## Introduction

Pulmonary artery aneurysm (PAA) is a rare clinical disease, with a prevalence rate of only 7.3/100,000, according to Deterling and Clagett autopsy statistics (1), and only a few cases have been reported domestically, especially in children. Reports of pulmonary artery aneurysm caused by infective endarteritis of the pulmonary artery attributed to patent ductus arteriosus in children are rare (2). The rarity of the condition leads to a general lack of knowledge on the part of pediatricians regarding the diagnosis and management of the disease, and currently, there are no diagnosis and treatment guidelines. In November 2022, we admitted a patient with pulmonary aneurysm formation following patent ductus arteriosus and infective endarteritis. This report summarizes the clinical data in order to promote awareness among pediatricians of this type of disease.

## Case description

**Figure 1:**
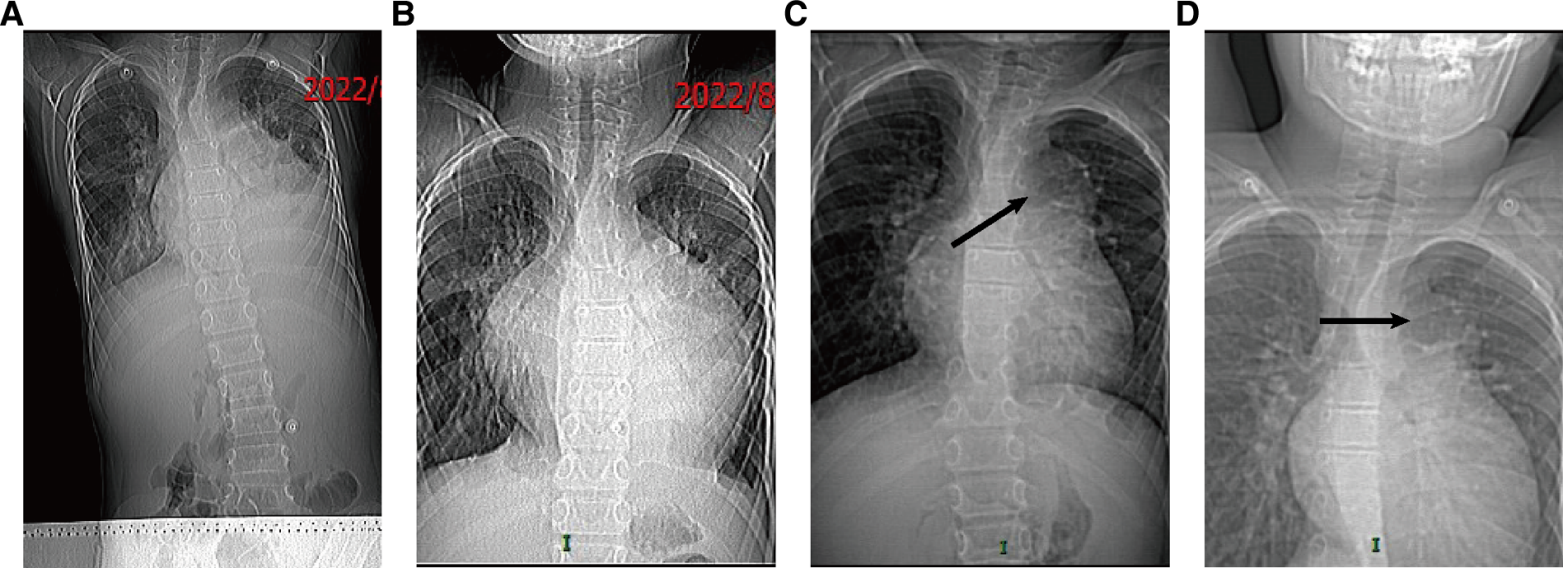
The follow-up chest x-ray from August to November: (**A**) chest x-ray on 10 August 2022; (**B**) chest x-ray on 21 August 2022; (**C**) chest x-ray on 16 September 2022; (**D**) chest x-ray on 14 November 2022.

**Figure 2:**
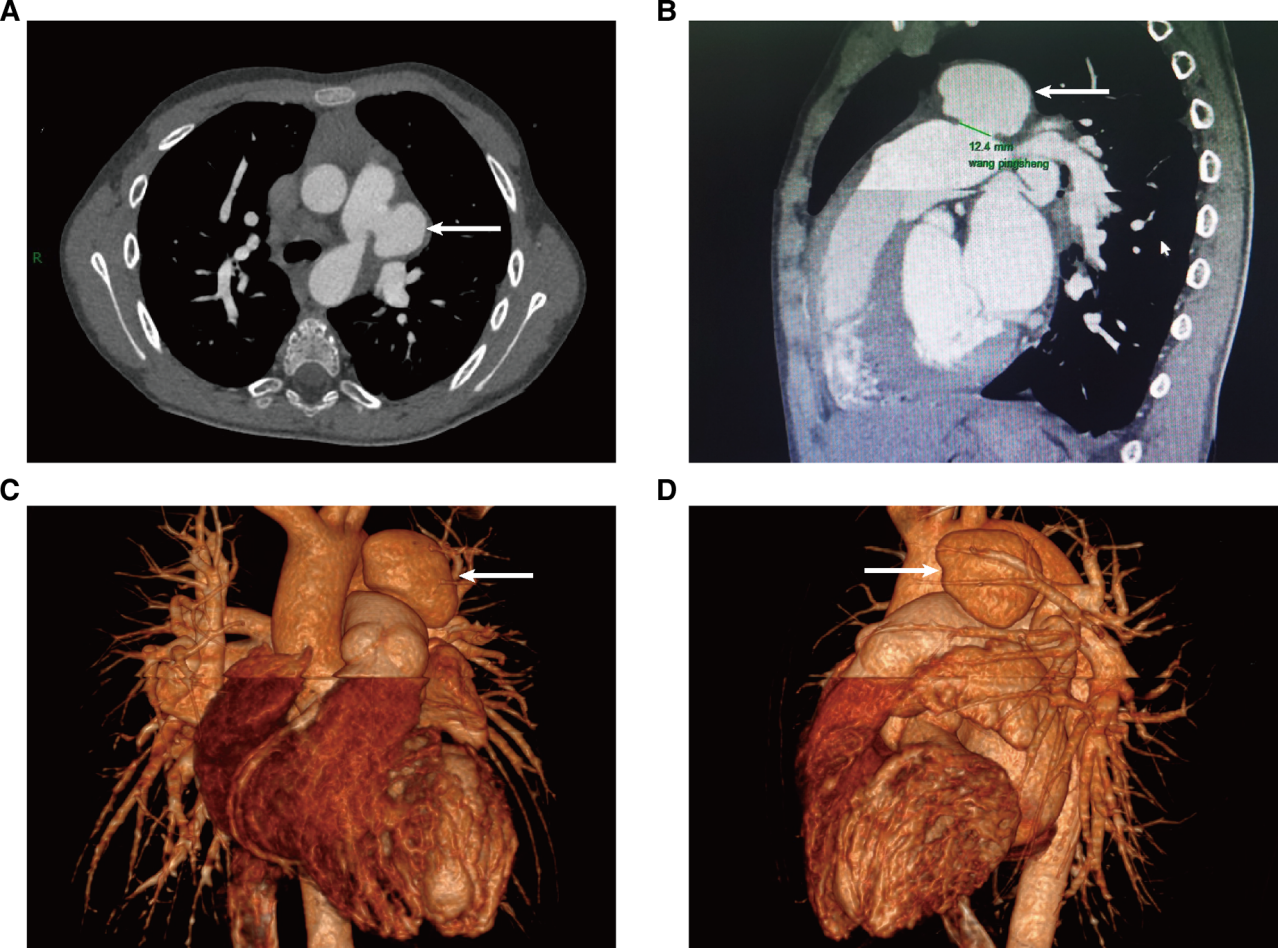
Cardiac contrast-enhanced CT on 14 November 2022; the PAA is indicated by the arrow. (**A**) Cross-section of CT images; (**B**) sagittal plane of a CT image; (**C,D**) 3D reconstruction of a CT image.

A 10-year-old boy was admitted to the hospital with a cardiac murmur for 8 years. The patient had no previous history of cyanosis, squatting, syncope, hemoptysis, chest pain, or recurrent pneumonia, and showed shortness of breath after activity since the age of 6. In August 2022, the patient was hospitalized in our department of cardiology with a diagnosis of “pneumonia, septic pericarditis, infective endarteritis, hydropericardium and pleural effusion”, and no pulmonary aneurysm was detected by cardiac ultrasound and chest CT several times during the hospitalization. The boy was diagnosed with infective endarteritis because of positive blood culture (micrococcus luteus), high white blood cell count (28.28 × 10^9^/l), and echocardiogram indicating vegetation in the left pulmonary artery. We administered anti-infective therapy according to the guidelines of infective endocarditis, waited for a negative blood culture result, followed by a full course of antibiotics for more than 4 weeks before proceeding with surgery. He was discharged after 17 days of anti-infective treatment and was continued on oral linezolid for 18 days. In September 2022, a follow-up chest CT showed a significantly enlarged cardiac shadow, and most of the pericardial and pleural effusions were absorbed. The follow-up chest x-ray from August to November is shown in Figure 1. The following were the results of the physical examination performed on admission: there was no differential cyanosis in the upper and lower extremities, thrill could be felt, persistent mechanical murmur could be heard between the second and third ribs at the left edge of the sternum, there was accentuation of P2 cardiac sound without fixed splitting, there were no clubbed fingers, and there was congenital absence of the left hand. An echocardiogram performed on 14 November 2022, revealed patent ductus arteriosus, with a left-to-right shunt flowing at 4.2 m/s; a mild tricuspid regurgitation flowing at 2.4 m/s; the size of the pulmonary artery was 31 × 19 mm, with an inner diameter of 9.8 mm; a strong echogenicity of 3 × 5 mm in size was detected at the opening of the left pulmonary artery. A cardiac CT angiography (CTA) revealed that the size of the pulmonary artery was 31 × 26 × 25 mm, and the end of the pulmonary artery of the patent ductus arteriosus was 5.8 × 4.8 mm (Figure 2). The electrocardiograph (ECG) showed sinus rhythm with high left ventricular voltage and electrical gradients in the QRS wave (QRS) wave group. Preoperative routine blood test, blood biochemical examination, CRP, hematocrit, syphilis screening, and tuberculosis screening showed no significant abnormalities.

**Figure 3:**
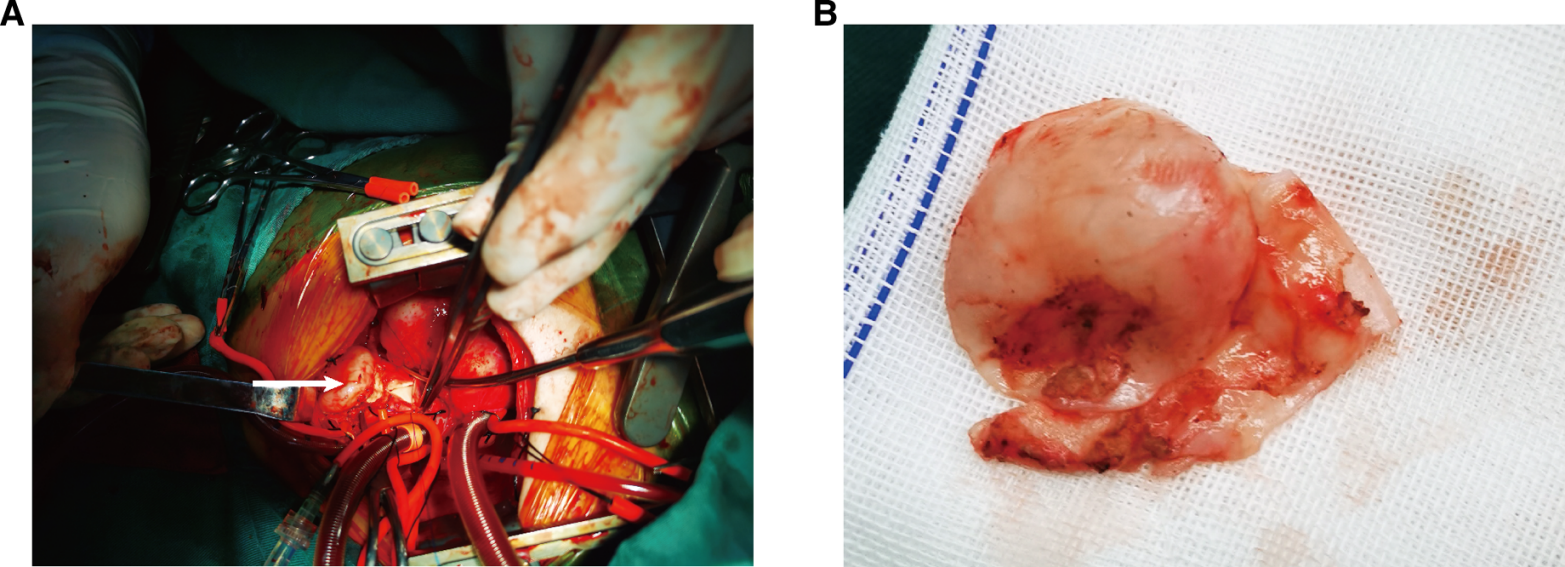
Surgical findings: (**A**) PAA bulge on the left side of the main pulmonary trunk (arrow); (**B**) appearance of aneurysm wall tissue.

We performed the surgery, which lasted for over 9 hours. We performed a median sternotomy and found severe pericardial adhesions; possibly the boy had pericarditis due to infective endarteritis. After instituting cardiopulmonary bypass, we freed along the distal pulmonary trunk, and its surrounding tissues were severely adherent and not easily identifiable. Thus, we decided to lower the anal temperature to 18°C in preparation for deep hypothermic circulatory arrest; the heart and cardiopulmonary bypass were arrested with del Nido cardioplegia through the aortic root. A longitudinal incision was made in the pulmonary trunk to identify the pulmonary artery end of the ductus arteriosus, the diameter of which was 10 mm. We used a bovine pericardial patch (Balance Medical) to repair the defect from the medial aspect of the pulmonary artery. After freeing the structures surrounding the pulmonary artery aneurysm, the aneurysm was seen to be approximately 30 mm in diameter and spherical in shape (Figure 3). A dissection of the aneurysm revealed no vegetations inside it or at the opening of the left pulmonary artery. After complete resection of the aneurysm, the pulmonary trunk was repaired with a bovine pericardial patch (Balance Medical) and continuous sutures with 5–0 Prolene sutures. The temperature was increased, the aorta was deaired, and the aortic clamp was removed. The anastomosis of the bovine pericardial patch oozed blood significantly, and repeated reinforcement and trimming of the pulmonary artery wall around the aneurysm proved ineffective. During the fourth aortic cross-clamp, a complete resection of the pulmonary aortic wall around the aneurysm was done, and a reconstruction of the pulmonary artery trunk with the bovine pericardium patch was carried out, following which the bleeding abated, the circulation finally stabilized, gauze compression was applied to stop bleeding, and the closure of the chest was delayed. The cardiopulmonary bypass time was 382 min. The aortic cross-clamp time was 60 + 36 + 2 + 69 min, 167 min in total. The deep hypothermic circulatory arrest time was 37 min. An intraoperative transesophageal echocardiography showed a patent pulmonary artery flow. After the operation, the patient was admitted to the cardiac intensive care unit (CCU), and the chest was closed successfully on the third postoperative day.

**Figure 4:**
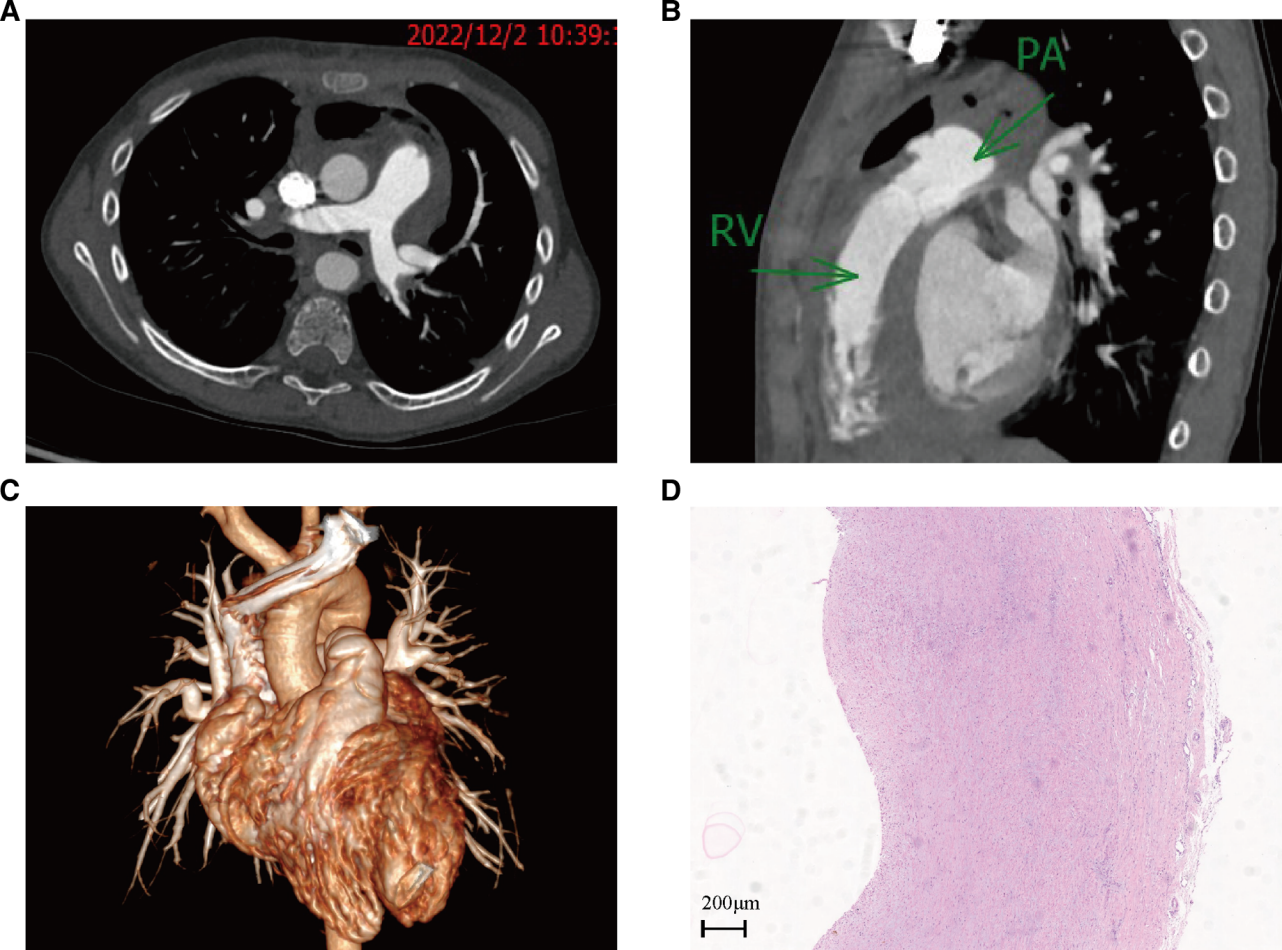
Cardiac contrast-enhanced CT on 2 December 2022, and HE-stained pathology of aneurysm wall tissue: (**A**) cross-section of CT images; (**B**) sagittal plane of a CT image; (**C**) 3D reconstruction of a CT image; (**D**) HE-stained pathology of aneurysm wall tissue.

The patient's surgery was successful with no serious complications. The postoperative cardiac CTA revealed that the pulmonary aneurysm disappeared, the pulmonary artery flow was patent, and the pulmonary artery trunk was significantly reduced (Figure 4). A pathological examination of the pulmonary artery aneurysm showed a vascular wall-like structure with a smooth inner layer and uneven thickness with varying degrees of fibrosis and mucoid degeneration, and a focal infiltration of acute and chronic inflammatory cells could be seen in the thick position. The boy reported no abnormal results on the postoperative follow-up echocardiogram at 1, 3, and 6 months. The CT scan also showed a smooth PA reconstruction and no aneurysm on the pulmonary artery.

## Discussion

Pulmonary artery aneurysm can be classified into proximal PAA and peripheral PAA depending on their location. Proximal PAA is used to describe aneurysms involving the pulmonary artery trunk and/or branch pulmonary arteries, 80% of which occurs in the pulmonary artery trunk. Peripheral PAA is used to describe aneurysms involving distal vessels (3). PAA is classified by etiology into congenital (patent ductus arteriosus, ventricular septal defect, and atrial septal defect) and acquired (syphilis, tuberculosis, atherosclerosis, and trauma) (1). The pathogenesis of an aneurysm is considered to be initiated by processes of injury and repair within the vascular wall. Congenital heart disease [patent ductus arteriosus (PDA) is the most common] is more susceptible to bacterial endocarditis, endovasculitis, or tricuspid valve endocarditis. Bacteria can directly invade or spread to the pulmonary arteries, which can lead to a degenerative necrosis of the middle elastic fibers and muscular layer of the arterial wall (4). The patient in our case had a proximal PAA that was caused by patent ductus arteriosus. The presence of a prolonged left-to-right shunt in the arterial duct created turbulence at the pulmonary artery end, where bacteria could easily colonize and thus induce bacterial endarteritis. Endarterial inflammation can lead to a degenerative necrosis of the middle elastic fibers and muscular layer of the arterial wall, which, together with a high-velocity shunt from the arterial duct impacting on the left lateral wall of the pulmonary trunk, can aggravate the damage to the vessel wall present there and eventually lead to acquired pulmonary aneurysm. Vasculitis has also been identified as an acquired cause of PAA (5).

The diagnostic gold standard of PAA is angiocardiography. However, an angiography can visualize only the patent lumen of the PAA, and it is invasive, expensive, and less common nowadays. With the development of CT technology, angiography is being gradually replaced. Enhanced CT allows multiplanar reconstruction, which, combined with echocardiography and MRI, can provide surgeons with highly accurate information. PAA is very rare and not well documented in books, appearing only in case reports. Many physicians lack awareness of PAA, such that missed diagnosis of the disease frequently occurs on imaging examination. A retrospective study showed that 46% of pulmonary artery pseudoaneurysms were overlooked by radiologists on the initial CT scans, all of which were contrast-enhanced studies (6). The fact that this patient's pulmonary artery aneurysm was not reported on multiple ultrasound and CT examinations before the diagnosis was confirmed indicated that a number of physicians had overlooked the imaging features of PAA, and more case reports were needed to provide experiences to minimize the omission diagnostic rate of PAA.

The clinical manifestations of PAA are atypical and varied, with hemoptysis, cough, or dyspnea as the initial symptoms. In our case, cough, fever, and dyspnea were the first symptoms. The development of PAA in our case showed that PAA can form in the short term after the occurrence of infective endarteritis, indicating that infection is a risk factor for PAA (7). On the other hand, PDA should be managed as early as possible to reduce the occurrence of PAA, provided that the indications for surgery are met. The appearance and the pathological report of the aneurysm showed that the wall of the aneurysm was thick and it would not rupture in a short time, but the risk of rupture will increase with time, especially when there is a non-uniform disruption in the arterial wall (8). According to Duijnhouwer's systematic review (9), pulmonary hypertension in congenital heart disease, fast PA diameter growth (>2 mm/year), and tissue weakness due to infection are high-risk factors for dissection or rupture of PAA, and therefore, we advocate early surgical management for children with congenital heart disease.

For the management of pulmonary aneurysms, both conservative and surgical treatments are available. A complete remission rate of 63.6% and an overall mortality rate of 18.2% were reported in patients who received only drug therapy (10). The development time of PAA in our case was short, and it could have been life-threatening in the event of dissection or rupture if it had not been operated in a time-bound manner. There are no clear guidelines on the indications for surgery in children, and therefore, we suggest that if PAA grows rapidly or is symptomatic, surgery should be performed soon after the diagnosis is made and if surgical contraindications are excluded. Radical surgery is preferred over surgical treatment, i.e., to correct the cause together with the aneurysm, either by aneurysmectomy with repair or by replacement of the pulmonary artery. Pulmonary arterioplasty can be performed using homogeneous arteries, artificial vessels, and an autologous pericardium; pulmonary artery replacement can be performed using artificial vessels or homogeneous external tubes (11). We used a glutaraldehyde-treated bovine pericardial patch to repair the pulmonary artery, the anastomosis of the bovine pericardial patch oozed blood significantly, and repeated reinforcement and trimming of the pulmonary artery wall around the aneurysm proved ineffective. During the fourth aortic cross-clamp, a complete resection of the pulmonary aortic wall around the aneurysm was carried out, and a reconstruction of the pulmonary artery trunk was done by using a bovine pericardium patch; the bleeding abated, and circulation finally stabilized. Repeated bleeding from the anastomosis is associated with infective endarteritis. Infection leads to inflammatory exudation of the pulmonary artery wall around the aneurysm, causing the artery wall to become lax and also cause recurrent bleeding after suturing, almost leading to fatal consequences. If a similar case is encountered, the pulmonary artery wall around the aneurysm needs to be removed and the main pulmonary trunk needs to be reconstructed. Hou et al. advocate that the most common procedure is the replacement of the pulmonary artery and the pulmonary trunk; this can be performed using Gore-Tex or Dacron tubes, homografts, or xenografts (10). In our patient, the pulmonary artery flow was patent postoperatively, and the patient recovered well.

## Conclusions

PAA caused by secondary infection of intimal inflammation of the pulmonary artery due to patent ductus arteriosus is rare and potentially fatal in children. Early diagnosis by echocardiography and contrast-enhanced CT is essential. PAA associated with anatomical anomalies, rapid growth, and compression of neighboring critical structures is a proper candidate for surgery. The surgical options include correction of anatomical anomalies, aneurysmectomy, and pulmonary arterioplasty/replacement. The surgical outcomes are satisfactory.

## Data Availability

The original contributions presented in the study are included in the article, further inquiries can be directed to the corresponding author.
